# “Hearken to the Hermit-Thrush”^1^: A Case Study in Interdisciplinary Listening

**DOI:** 10.3389/fpsyg.2020.613510

**Published:** 2020-12-09

**Authors:** Emily L. Doolittle

**Affiliations:** Department of Research and Knowledge Exchange, Royal Conservatoire of Scotland, Glasgow, United Kingdom

**Keywords:** zoomusicology, ecomusicology, blackbird, hermit thrush, pentatonic scale, birdsong, music, music composition

## Abstract

Birdsong is widely analysed and discussed by people coming from both musical and scientific backgrounds. Both approaches provide valuable insight, but I argue that it is only through combining musical and scientific points of view, as well as perspectives from more tangentially related fields, that we can obtain the best possible understanding of birdsong. In this paper, I discuss how my own training as a musician, and in particular as a composer, affects how I listen to and parse birdsong. I identify nine areas of overlap between human music and birdsong, which may serve as starting points both for musical and scientific analysis, as well as for interdisciplinary analysis as practiced in the developing field of “zoomusicology.” Using the hermit thrush (*Catharus guttatus*) as an example, I discuss how the song of a single species has been described by writers from a variety of disciplines, including music, literature, and the sciences, as well as how we can contextualise these varied perspectives in terms of broader cultural thought trends. I end with discussion of how combining approaches from multiple fields can help us to figure out new questions to ask, can help us identify how our own cultural biases may affect how we hear birdsong, and ultimately can help us develop richer and more nuanced understandings of the songs themselves.

## Introduction

Musicians and ornithologists alike have a long history of listening closely to, analysing, and writing about birdsong, but they typically do so in very different ways. Musicians are more likely to focus on the subjective or visceral experience of hearing the song, or to look for points of comparison with human music. Scientists, on the other hand, tend to focus on objective description, gathering quantifiable data, and finding functional explanations for song structure and singing behaviour. Both approaches provide valuable insight, but I argue that it is only through combining musical and scientific points of view, as well as perspectives from more tangentially related fields, that we can obtain the best possible understanding of birdsong. In this paper, I discuss how my own training as a musician, and in particular as a composer, affects the way I listen to and interpret the songs of birds and other non-human animals. I identify nine areas of potential overlap between human music and birdsong, which can be used as starting points for both musical and scientific analysis, as well as for interdisciplinary analysis, as practiced in the developing field of “zoomusicology.” Finally, using the hermit thrush (*Catharus guttatus*) as an example, I discuss how the song of a single species can be understood from a variety of perspectives, including musical, poetic, naturalistic, and scientific, as well as how we can contextualise these perspectives in terms of broader cultural thought trends. I end with discussion of how combining approaches from multiple fields can help us to figure out new questions to ask, can help us identify how our own cultural biases may affect how we hear birdsong, and ultimately can help us develop richer and more nuanced understandings of the songs themselves.

**Figure 1:**
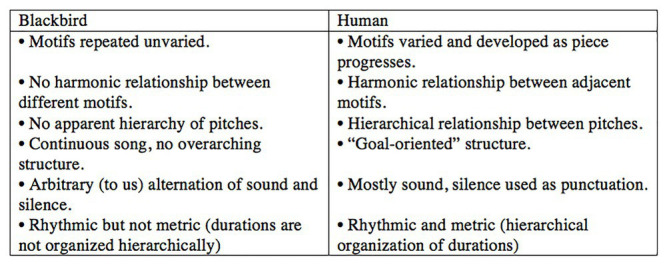
Blackbird-like vs. human-like arrangements of motifs. Figure adapted from *Music Theory is For The Birds* (Doolittle, 2015).

I have been interested in birdsong since 1997, when I heard a Eurasian blackbird (*Turdus merula*) for the first time, shortly after I had moved from Canada to the Netherlands to study music composition. I was fascinated by the way small bits of what it sang sounded a lot like human music – clear, pure notes, repeated phrases, scale‐ and arpeggio-like passages, and so on – even though the structure of its song as a whole was very different than any of the human musical forms I was aware of. I decided to explore blackbird song through writing a piece of music based on a series of blackbird motifs (short sequences of sounds): I would arrange these motifs first in a blackbird-like way, and then gradually transform their organisation into something more typical of the human music I knew. I began by transcribing the song of the blackbird outside my window into musical notation and making a list of the differences between the ways, I thought the blackbird and a human might arrange the same collection of motifs. Here are a few (Figure 1).

At that time, I was studying with the composer Louis Andriessen. When I showed him my list, he looked at the blackbird column and said “that sounds like Stravinsky!” And of course, he was right that while blackbird song might not sound much like the “common practice era” music (Western classical music written between 1600 and 1900) that still makes up the bulk of a classical music education, there are many kinds of music that do sound like birdsong (Mâche, 1983/1992; Head, 1997; Martinelli, 2002, 2008; Rothenberg, 2005, 2019; Bowden, 2008; Doolittle, 2008, 2015; Taylor, 2011, 2017; Feld, 2017, and others). Indeed, it has been speculated that Igor Stravinsky’s distinctive style of juxtaposing contrasting blocks of material without using developmental or connecting passages, as exemplified in *The Rite of Spring* (1913), was influenced by the songs of birds native to Ustilug (Ukraine), where he spent his summers (Mâche, 1983/1992, p. 121).

As I was working on my piece, the bird which had initially attracted my interest left my neighbourhood and was replaced by a (presumably) younger blackbird with a simpler, more repetitive song that would not have been at all suitable for my piece. To finish it, I had to transcribe the song of the blackbird in a friend’s garden instead. That song was just as interesting as, but completely different from, the song of the original blackbird. I had not known that the songs of different birds of the same species could vary so much. I started reading ornithological literature and learned about vocal learning, imitation, and dialects in songbirds (known scientifically as oscine passerines). Though the sound and motivic structure of Eurasian blackbird song is what had initially piqued my musical interest, it turned out there were behavioural and cognitive similarities between music and birdsong too. Birdsong, like my own composing, was a cultural activity (Slater, 1986).

## Zoomusicology

Of course I am not the first person to be interested in the relationship between animal songs and human music. Humans have incorporated the songs and calls of non-human animals into their music for as long as we have oral and written records (Mâche, 1983/1992; Gioia, 2006; Levin and Süzükei, 2006; Doolittle, 2008; Rothenberg, 2016, 2019). Similarly, there is a long history of describing animal songs using human musical terms (Kircher, 1650; Barrington, 1773; Hawkins, 1776; Catchpole and Slater, 1995; Head, 1997; Baker, 2001; Mundy, 2018). Some of these comparisons seem superficial: a birdsong may have a “flutey sound,” but so does a tea kettle whistling, which would only be considered “music” under the broadest of definitions (Doolittle, 2007, p. 63). Others are wrong. For example, Canyon Wrens (*Catherpes mexicanus*) are frequently described as singing “in the chromatic scale” (Baptista and Keister, 2005; Hartshorne, 1973, p. 84), which they do not.^2^ But at their best, comparisons may point to deep-seated similarities in structure or function. Indeed, a growing body of zoomusicological literature explores these similarities.

The term “zoomusicology” (“*zoomusicologie*”), typically defined as the study of the “aesthetic use of sound communication among animals” (Martinelli, 2002, p. 7), was popularised by French composer François-Bernard Mâche in his 1983 work *Musique*, *mythe*, *nature*, *ou*, *les dauphins d’Arion*. A highly interdisciplinary field, zoomusicology draws on research practices and analytical techniques from music, philosophy, and the sciences alike to better understand the songs of non-human animals, as well as human musical practices which may be related to or influenced by them. Currently active zoomusicologists include Martinelli (2002, 2008), who draws on his background as a musicologist and semiotician to develop zoomusicological methodologies; Taylor (2011, 2013, 2017), who brings her experience as a composer and performer to a long-term study of the song of the pied butcherbird (*Cracticus nigrogularis*), and of animal musicality in general; Rothenberg (2005, 2010, 2013, 2016, 2019), who explores the connection between non-human animal songs and human music through philosophical writing and performance (Rothenberg et al., 2014); and South (2020), who combines musical and scientific approaches to the study of humpback whale (*Megaptera novaeangliae*) song. Introductions to zoomusicology as a field include texts and online resources by Mâche (1997), Martinelli (2001), Taylor (2012a,b), Keller (2012), and Doolittle and Gingras (2015). Related research may also sometimes be described as “biomusicology” (Wallin et al., 1999), “ecomusicology” (Allen, 2011), or “multispecies ethnomusicology” (Silvers, 2020), or conducted under the auspices of more traditional academic fields.

Though there is no universally agreed upon definition of music, and no universal criteria for an animal song to be considered “aesthetic,” there are many points of potential overlap between human music and non-human animal song.^3^ The following are some that I find particularly compelling (labelled for reference in subsequent sections of this paper).

## Areas of Overlap Between Non-Human Animal Song and Human Music

**a. Vocal learning:** Humans are born with the ability to learn to make music (and speech) from other humans. Vocally learning birds [songbirds (oscine passerines), parrots, and hummingbirds; Catchpole and Slater, 1995], as well as the small number of vocally learning non-human mammals (including whales and dolphins, bats, seals, and elephants), must also learn their songs from other members of their species (Janik and Slater, 1997; Deecke et al., 2000; Rendell and Whitehead, 2003; Poole et al., 2005; Janik, 2014; Knörnschild, 2014). By contrast, non-vocally-learning species are born with the ability to develop their expected species vocalisations without needing exposure to other members of their species (Kroodsma and Konishi, 1991).
**b. Culture:** Vocally learning species learn the music/vocalisations of conspecifics around them, rather than of their genetic ancestors. For example, just as humans learn the music they hear or are taught, songbirds of the same species in different regions may sing different versions of their songs, called “dialects” (Catchpole and Slater, 1995, p. 193–218). Humpback whales in the same ocean basin usually sing the same, constantly changing song, though they also occasionally have song “revolutions” in which they learn a new song from neighbours or from conspecifics from another ocean basin (Noad et al., 2000; Garland et al., 2017).
**c. Pattern creation:** Both humans and non-humans may use fixed or variable sequences of pitches, intervals (ratios between adjacent pitches), timbres, durations, and other sound elements to create recognisable motifs or phrases, which may recur within or between pieces/songs, or may be varied throughout the piece/song (Nettl, 1999; Baptista and Keister, 2005; Doolittle, 2007, p. 161–189).
**d. Structure/Form:** Human music often fits into culturally-determined structures: one example is “sonata form,” which was common in late 18th‐ and 19th-century Europe (Rosen, 1980). Similarly, different species may have characteristic song structures. For example, plain-tailed wrens (*Thryothorus euophrys*) sing in repeated four-part form (ABCD), with males singing the A and C parts and females the B and D parts (Mann et al., 2009, p. 21). Other species, like the sedge warblers (*Acrocephalus schoenobaenus*), continually order and reorder a fixed or variable set of motifs, more akin to improvisatory forms of human music (Beecher and Brenowitz, 2005).
**e. Individual variation within a given style:** Two human musicians trained in the same tradition and performing the same piece will nonetheless perform it differently. In many vocally-learning non-human species, individuals are also readily distinguishable, though species-specific stylistic patterns, such as number of repeats, types of sounds used, range, timbre, ratio of sound to silence, and so on usually make the species readily identifiable (Mâche, 1983/1992, p. 141; Martinelli, 2008, p. 140–158).
**f. Functional identity with non-functional stylistic change:** While variation sometimes exists only in an individual song, it may also lead to change over time. According to cultural theorist Peckham (1967, p. 71), one sign that an object may be aesthetic is if “a chronologically arranged sequence of such objects shows both functional identity and non-functional stylistic dynamism.” In human music, an example might be the continual variation in the style of orchestral music over the past 200 years, while the context – an evening performance in a concert hall – has remained largely the same. Humpback Whale song, too, continually changes, but its context – performed by males, primarily during breeding season – stays constant (Payne et al., 1983; Noad et al., 2000; Garland et al., 2017).
**g. Play with pattern and surprise:** Philosopher and ornithologist Hartshorne (1956) proposed the idea of a “monotony threshold”, hypothesising that animal songs need a certain amount of variety to keep the listener engaged. He theorised that aesthetic qualities arise in both human and non-human song as the singer strives to find the right balance between “expected repetition and the unexpected, that joint avoidance of monotony and chaos on a sufficient level of complexity, which is beauty” (Hartshorne, 1973, p. 9). The unexpected may be heard in relation to what has come previously in that piece or song, or in relation to what is expected for that musical style, or species, or both (Goehr, 1990, p. 141).
**h. Connection with physical and/or emotional state:** The music we make varies according to activity and emotion: for example, a lullaby is likely to sound different than a dance (Mehr et al., 2017, p. 256–268). Similarly, non-human animal vocalisations vary according to context (such as whether they are singing to young in the nest or to rivals) and emotional state (such as whether they are calm or aggressive; Martinelli, 2008, p. 126–127).
**i. Singing for intrinsic reward:** Musicians have long suspected that some non-human species may sing not only for functional reasons like attracting mates, defending territory, or reinforcing group bonds, but also for enjoyment. Whereas we have previously only been able to infer this from observation of singing behaviours or through comparison of animal song structures with human musical structures, recent research on the connection between singing behaviour and hormones in starlings (*Sturnus vulgaris*) supports the idea that some non-human species, too, may sing in part for “intrinsic reward” (Kubikova et al., 2010; Rothenberg et al., 2014; Stevenson et al., 2020).

Of course this is only a partial list, and others might emphasise other aspects – for example, Martinelli (2008, p. 133–214) gives a comprehensive list of “zoomusicological universals” in *Of Birds, Whales, and Other Musicians*, while Taylor (2013) emphasises the importance of comparing not only sound objects, but also music-related activities, such as warming up, practicing, improvising, or composing. When I began researching animal song, I wanted to convince everyone that some non-human animal songs should be considered music, but I no longer feel this is important – or even possible. What is clear is that there are enough similarities between some non-human animal songs and some human music that we can use some of the same perceptual skills and theoretical tools to look at both (Doolittle, 2007, 2015).

## The Hermit Thrush

**Figure 2:**
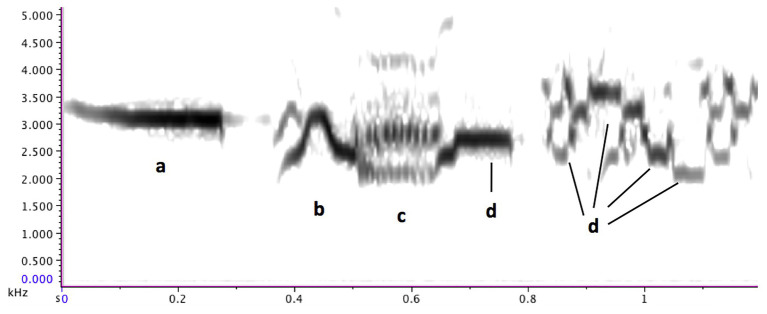
A hermit thrush song-type. **(A)** introductory whistle, **(B)** slide, **(C)** trill, and **(D)** some steady-pitch notes. Figure reprinted from *Music Theory is For The Birds* (Doolittle, 2015).

The hermit thrush is a small (14–18 cm) North American songbird with a song that is widely considered beautiful (Rothenberg, 2005, p. 116; Doolittle, 2020). They live in a wide range of habitats, including boreal forests, deciduous woods, and mountain forests, across southern and central Canada, the United States, and northern Mexico, making them one of the commonest songbirds, though their reclusive nature means that people are not always aware of their presence (Cornell Lab of Ornithology, 2019). Each male sings 7–12 different “song-types,” with several seconds of silence following each song-type **(d)**. Each song-type consists of a long “introductory whistle,” followed by one or two phrases of cascading shorter notes. The song may also contain some trills, multi-pitches, and noise sounds, but clear notes with a measurable pitch (“steady-pitch notes”) predominate (**c, d, e;** Roach et al., 2012; Doolittle et al., 2014). Song-types are highly stereotyped, though occasionally a segment of the final phrase may be repeated (**d, e, g;** Figure 2).

Song-types are presented with immediate variety in a semi-fixed, semi-unpredictable order, reminiscent of a second order Markov chain (**d, e, g;** Dobson and Lemon, 1977; Roach et al., 2012), with songs with a higher frequency introductory whistle typically alternating with songs with a lower frequency introductory whistle (**c, d;** Kroodsma, 2005, p. 262–266). There are minor differences in song structure between eastern and western populations of hermit thrushes, with the average introductory whistle of eastern birds being lower in frequency and shorter in duration than the average introductory whistle of western birds (Roach and Phillmore, 2017). These geographical differences in song structure, combined with song-learning studies in the closely related wood thrush (*Hylocichla mustelina*; Lanyon, 1979), suggest that hermit thrushes, like other songbirds, learn at least certain aspects of their songs from conspecifics (**a, b;** Roach and Phillmore, 2017). Lack of the sharing of specific song-types between neighbouring birds (Jones, 2005; Roach et al., 2012), however, suggests that while the overall song structure may be learned, there may also be individual variation in the details of the cascading phrases (**a, d**, and **e**). Further research is needed to determine whether or not there are adaptive reasons for the divergent song characteristics between eastern and western populations, and whether these differences are perpetuated genetically or culturally (Roach and Phillmore, 2017). If these song differences turn out to be culturally perpetuated and non-adaptive, this raises the intriguing possibility that aesthetic preference, whether on the part of the male singers, the female listeners, or both, might be a driving force behind the different eastern and western “styles” of hermit thrush song (**f**).

Naturalists, musicians, poets, and scientists have all written extensively about the hermit thrush since the early 1800s^4^. As such, hermit thrush song provides a perfect case study into both the insights and the limitations of each of these perspectives. For the first half of the 19th century, the European explorers and settlers who wrote these guides were unaware of hermit thrush song. But as settlers and their descendants became more deeply embedded in their surroundings, they learned that the hermit thrush not only sang, but sang beautifully. Whereas the “Father of American Ornithology” Alexander Wilson could write in 1831 that the hermit thrush made only an “occasional squeak,” by 1840, English-American zoologist Thomas Nuttall described the bird as “scarce inferior to (the Nightingale) in its powers of song” (Coues, 1878, p. 33–34).

Catskills-born naturalist John Burroughs (1835-1927), whose seminal “nature essays” were formative in the developing American conception of nature and the wild (Walker, 1997, p. 278), gave the hermit thrush a central role in his writings:

This song appeals to the sentiment of the beautiful in me, and suggests a serene religious beatitude as no other sound in nature does. “O spheral, spheral!” he seems to say; “O holy, holy! O clear away, clear away! O clear up, clear up!” interspersed with the finest trills and the most delicate preludes. It is not a proud, gorgeous strain. Suggests no passion or emotion, – nothing personal, – but seems to be the voice of that calm, sweet solemnity one attains to in his best moments. It realises a peace and a deep, solemn joy that only the finest souls may know. Listening to this strain on the lone mountain, with the full moon just rounded from the horizon, the pomp of your cities and the pride of your civilisation seemed trivial and cheap (Burroughs, 1866, p. 51).

In this, as well as in many similar passages published between then and his death in 1921, Burroughs emphasised the hermit thrush’s reclusive nature (“on the lone mountain”), the music-like beauty of its song (“the finest trills and the most delicate preludes”), and the song as a potential conduit to the holy (“a serene, religious beatitude”).

Burroughs became friends with poet Walt Whitman (1819-1892) in 1865, and shared with him his enthusiasm for the hermit thrush. Whitman made prominent reference to hermit thrush song in his poems *When Lilacs Last in the Dooryard Bloom’d* (1866) and *Starting from Paumanok* (as published in the 1867 version of *Leaves of Grass*), using imagery borrowed directly from Burroughs. The bird is solitary: “The hermit withdrawn to himself, avoiding the settlements” (Burroughs, 1866, p. 4). It is a remarkable singer: “O wondrous singer” (Burroughs, 1866, p. 11). And its song can connect us with the divine: “Covering the earth and filling the spread of the heaven/As that powerful psalm in the night I heard from recesses” (Burroughs, 1866, p. 16). Together, Burroughs and Whitman popularised not only awareness of hermit thrush song, but also of a particular set of symbolic values to ascribe to it. This transcendentalism-tinged symbolism suited the zeitgeist of late 19th and early 20th century English-speaking North America: between the 1860s and 1940s, hermit thrush song featured in no less than 128 poems, including the poem of Cheney (1905), which gives this essay its title, as well as in dozens of nature essays by then-well-known writers, including Bolles (1891), Tolman (1900), and Kirkham (1908), and a pair of piano pieces by American composer Amy Beach (Von Glahn, 2013, p. 40–46; Mundy, 2018, p. 33–34; Doolittle, 2020).

Late 19th and early 20th century poetic and naturalist writing about the hermit thrush were thus deeply intertwined. But while poets typically stuck to emotive and symbolic representation of hermit thrush song, those writing nature essays and guides often attempted to record and communicate the sound of the song itself. For this, they turned to musical terminology, in what might retroactively be seen as some early essays into zoomusicology. In *Wood Notes Wild*, musician-turned-naturalist Cheney (1892, p. 59–60) used Western music theoretical terms to describe the intervals (ratios between adjacent pitches) and rhythmic patterns in hermit thrush song: “The hermit, after striking his first low, long and firm tone, … bounds upwards by thirds, fourths, fifths, and sometimes a whole octave, gurgling out his triplets with every upwards movement.” Cheney clearly understood the structure of hermit thrush song, and used the most accurate language available to him to describe it. Though “third,” “fourth,” “fifth,” “octave,” and “triplet” are terms borrowed from Western music theory, Cheney simply used them descriptively to refer to the frequency ratios 6/5 or 5/4, 4/3, 3/2, and 2/1, respectively, as well as to three-note rhythmic groupings: he did not try to fit hermit thrush song into any Western classical musical forms or structures. But other writers not only described hermit thrush song using Western theoretical terminology, but also ascribed to it the tonal hierarchies and structures of Western classical music. Historian Smith (1903, p. 372), for example, noted that the “keys” of the Hermit Thrush’s song “form part of the scale of A flat major.” Mathews (1904, p. 241), author of a popular series of field guides, including *Field Book of Wild Birds and Their Music*, believed that the hermit thrush had a sense of harmonic progression. “Not content with a single key, he deliberately chose several in major and minor relationship….” He went on to compare hermit thrush song to Beethoven’s *Moonlight Sonata*, as well as to passages from Wagner and Strauss (240–244). Biologist Oldys (1913, p. 540) heard complex harmonic relationships, describing one song that “…completely satisfies the requirements of human music… (with) a very attractive, and at the same time a very human, harmonic progression – from B to E minor, then to A with the minor seventh (C# being the basal note), which leads naturally into D.” Lest we be tempted to read these musical analyses as merely metaphorical, Oldys (1913, p. 541) stated directly that “in the case of the Hermit Thrush, we must discard the untenable theory of coincidence and declare that the bird expresses itself in human music.”

From today’s vantage point, most would agree that although a birdsong might sound like human music – and might even be its own kind of music – it is vanishingly unlikely that it would be organised according to the principles of Western classical music, or indeed the music of any specific human culture (Kivy, 1990, p. 24). Even two kinds of human music that sound superficially similar may be conceptualised completely differently. Unfortunately, as researchers began to realise the unlikelihood of hermit thrush song being structured according to the principles of Western classical music, this idea was supplanted with an even more troubling set of beliefs: that animal song must be equivalent to “primitive” human music (Sully, 1879, p. 619; Weismann, 1890, p. 358; Wallaschek, 1893, p. 237–241; Roberts, 1932; for further reading see Mundy 2018); that the pentatonic (five note) scale was “the musical mode instinctively adopted by primitive man” (Hopkins, 1922); and that hermit thrushes – regarded by many as the finest North American singer (Minot, 1880) – must therefore sing in the pentatonic scale. This is problematic on multiple fronts: no people are “primitive”; no extant music is “primitive” (and we have no way of knowing what the music of the first humans was like); even if there were such a thing as “primitive” human music there is no reason to believe a bird would sing it; pentatonic scales are by no means universal in human music, “primitive” or otherwise^5^; and, as will be shown, hermit thrushes do not sing in pentatonic scales!.

Successive versions of Mathews’s *Field Book of Wild Birds and their Music* provide clear evidence of how belief informed the supposedly objective finding of pentatonic scales in hermit thrush song. Whereas in the 1904 version of *Field Book* Mathews enthusiastically described the song in terms of the major and minor scales of Western classical music (see above), in the preface to the 1921 version he stepped back from his earlier analysis. “It may seem rather extravagant praise to sum up the song of the Hermit Thrush in the unqualified terms I have used on pages 256–257…” (Mathews, 1921, p. xxxvii), and went on to reinterpret the song as being based on pentatonic scales, which he calls the “primitive mode common to all folk-song” (xxxviii). “To sum it up in a few words, no other bird has developed what is plainly an intelligent use of a musical scale aptly fitted for expressive song – the so-called Pentatonic Scale.” And later: “Our Hermit has not progressed beyond that quaint primitive scale; just there he has reached his own limitation, why should we expect more?” (xxxix). Whereas Mathews had previously compared hermit thrush song to the works of canonical Western classical composers, Beethoven, Strauss, and Wagner, he now compares it to the music of the “Scotch piper” and the “southern Negro” (Mathews, 1921, p. xxxix).

**Figure 3:**
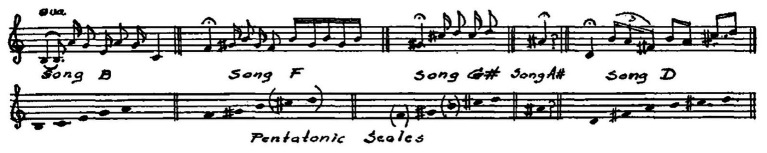
Wing’s transcription of five hermit thrush songs, and the pentatonic scales she believes them to be based on (Wing, 1951).

**Figure 4:**
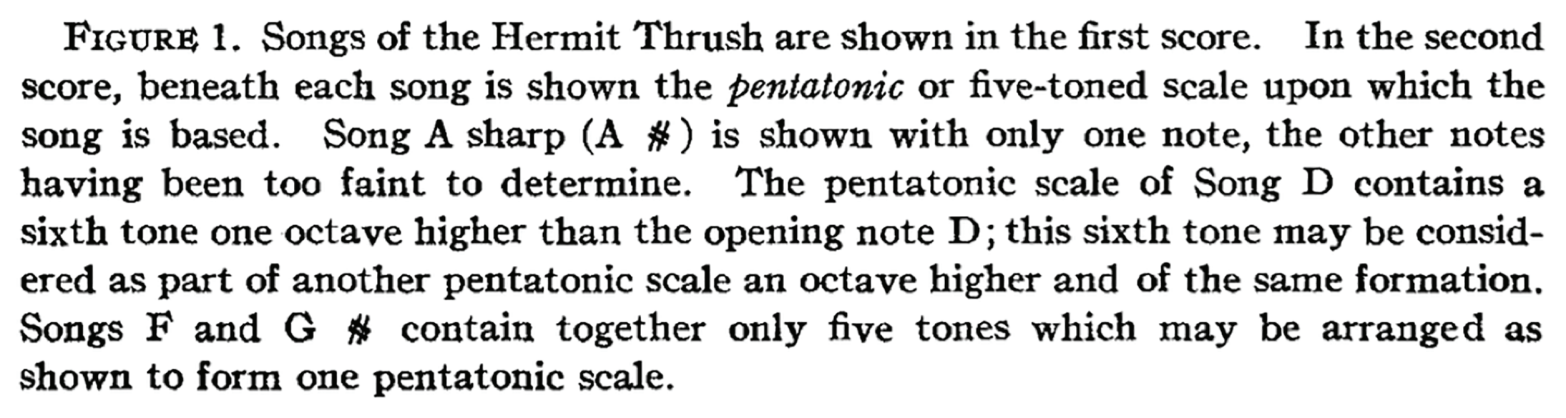
Wing’s justification of the pentatonic scale as the basis of hermit thrush song (Wing, 1951).

Naturalist Wing (1951), inspired by Mathews’s idea that hermit thrush songs were based on the pentatonic scale, provided the most extensive attempt at a “proof” of this theory, in her otherwise keenly observed *Notes on the Song Series of a Hermit Thrush*. Wing’s transcription of the song of a single hermit thrush (Figure 3) may well be accurate enough (insofar as pitches can be perceived reliably by ear at full speed and transcribed precisely within the constraints of Western music notation). But her argument that the song is based on the pentatonic scale is convoluted and unconvincing at best (Figure 4).

Even setting aside the questions of whether just any collection of five notes can be considered a “pentatonic scale,” or why a hermit thrush would follow a culturally-specific human music theory, most of these songs do not even have five notes in them. One uses six pitches (though this does include an octave repetition), two use three pitches, and one was un-notable, leaving only one that actually uses five notes. The idea that hermit thrush song was based on the pentatonic scale comes not from observation, but from ideology. As ornithologist Kroodsma (2005, p. 268–270), who is similarly skeptical of the hermit thrush pentatonic theory, puts it, this is a prime example of the maxim “I would not have seen it if I had not believed it.”

A book preface from 1921 or a one-off paper from 1951, which misinterpret hermit thrush song would be little more than a historical curiosity, had they not somehow captured the imagination of not only the general public, but also of numerous subsequent researchers. To give just a few examples, A. A. Hopkins repeated Mathew’s belief that hermit thrushes sing pentatonic scales in a 1922 Scientific American article, claiming that “The hermit thrush has the distinction, above all other birds, of having developed the Scotch, or pentatonic scale… the musical mode instinctively adopted by primitive man,” and Wing’s research was cited in work by Halafoff (1968), Ogborn (1976), Hartshorne (1973, p. 95), Gray et al. (2001), and Baptista and Keister (2005). The article by Gray et al. (2001), *The Nature of Music and the Music of Nature*, was published in the prestigious and widely read journal *Science*, lending new credibility and visibility to the myth of the hermit thrush pentatonic scale. Subsequent writings such as articles by Natalie Angier (2001) and Bossomaier and Snyder (2004), attribute the pentatonic hermit thrush song idea to Gray et al. (2001), without reference to Wing’s original paper (or to the Mathews’s preface which inspired it). Numerous popular books and web pages, including the Cambridge Dictionary online, now simply state that the “hermit thrush uses a pentatonic scale” (Cambridge Dictionary Online, 2020) without attribution. The hermit thrush pentatonic theory is now thus “common knowledge,” even though there has never been any credible evidence that this is true.

It is not surprising that European and North American writers before the mid-20th century used Western classical music theory as a framework for understanding birdsong. Lack of recording equipment meant they had to make on-the-spot analyses based on one-time hearings of the songs, and an anthropocentric and Eurocentric worldview was so deeply ingrained as to be invisible to its adherents. Those who understood the world from a non-Eurocentric point of view would have had less access to publishing, especially in a scientific or scholarly context. These early naturalists mapped what they heard on to what they knew, and described the song as accurately as they could with the limited set of tools and contextual knowledge available to them. But as mid-century technological advances began to allow repeated listening, listening to the song in smaller sections and at reduced speed, visualisations of sound, and more objective measurement, and as researchers began to understand that a Western outlook was not the only one possible, the belief that hermit thrush song should be based on either Western classical music or its imaginary other, “primitive” music, became increasingly untenable.

Perhaps in an effort to avoid the now-glaring errors of anthropomorphism and Eurocentrism made by earlier writers, recent scientific writing about hermit thrush song has tended to avoid substantive musical comparisons (though the timbre is sometimes referred to as “flutey” or “flute-like”). The following descriptions are typical:

Each song began with the characteristic introductory whistle, followed by a distinctive, complex series of fluty warbles (Rivers and Kroodsma, 2000, p. 468).
Their song frequency band was 1.4–8.6 kHz… The song begins with an introductory note… followed by a series of flute-like body notes. Many songs also contain structures like the upsweeps. The bird… had an 11-song repertoire that contained about 350 notes and structures (i.e., upsweeps and downsweeps; Jones, 2005, p. 14–15).
Overall, introductory note frequencies among all song types observed ranged from 1,617–5,062 Hz. However, in each population there was a distinct gap in the distribution of introductory note frequencies (ranging between about 3,000 and 3,400 Hz), such that introductory note frequencies were not normally distributed throughout their range (Roach et al., 2012).

These descriptions are objectively true, but are also less evocative of the sound and structure of the song than the earlier descriptions which fit it into a human musical framework. They also serve to distance us from our emotional, visceral, and aesthetic reactions to hearing birdsong and from our sense of connection to other species. As Taylor (2017, p. 37) puts it, “Despite the best effort of their authors, dry facts convey *more* than just facts. Metaphor is language’s essential anchor, and thin language distances us from animals, impoverishes our understanding of animal abilities, and disguises how songbirds serve the human imagination.”^6^ Kroodsma (2005, p. 255–267), alone among the modern ornithologists, does write about the music-like aspects of hermit thrush song in his general readership book *The Singing Life of Birds*, published after his retirement from academia, but this kind of musical comparison never makes it in to his scientific publications.

Abandoning inappropriate frameworks for understanding birdsong is essential if we wish to understand the songs on their own terms. But to ignore entirely our subjective apprehension of the songs is to discard one of the most important tools, we have for making sense of the world around us. We can never know exactly how a bird thinks about or hears its own songs, but as living beings with the ability to make and interpret our own complex vocalisations, we may be able to bring insight that is lacking in purely objective analyses. Indeed, automated categorisation of sounds is still calibrated against human aural and visual categorisation as a measure of accuracy (Deecke and Janik, 2006). Though the history of interpreting birdsong according to the structures of Western classical music is both fascinating and problematic, there is nothing inherently Western-centric about looking at the specifics of pitch relationships, rhythmic relationships, timbre, and/or the patterns created by these relationships. Many kinds of music worldwide give patterns and the relationships between patterns an organisational role and musicians may be ideally placed to perceive music-like patterns and structures in the songs of other species. If I approach my own zoomusicological research from the point of view of someone trained in Western classical music, this is only because it is the kind of music I know best, not because it is inherently any more likely to provide zoomusicological insight than any other kind of music.

In 2008, I embarked on a collaborative research project with evolutionary and cognitive biologist (and accomplished amateur musician) Tecumseh Fitch, music theorist, and cognitive biologist Bruno Gingras, and computational psychologist Domink Endres (Doolittle et al., 2014). We wanted to understand the structure of hermit thrush song, as well as why it has been such an enduring source of fascination for human listeners, but we found the earlier theories unconvincing and improbable and the more recent scientific analyses incomplete. Our intent was to bring together the subjective perception of relationships and patterns that is typical of musical analysis (Cook and Clarke, 2004, p. 3–14), with the more objective measurement tools, statistical methods, and predictive modeling techniques that are essential in scientific analysis. We strove to be aware of any cultural biases we had that might affect how we understood the song, while also recognising that it would be impossible for us as researchers to be entirely uninfluenced by (or even fully aware of) the cultural milieu in which we operate.

**Figure 5:**
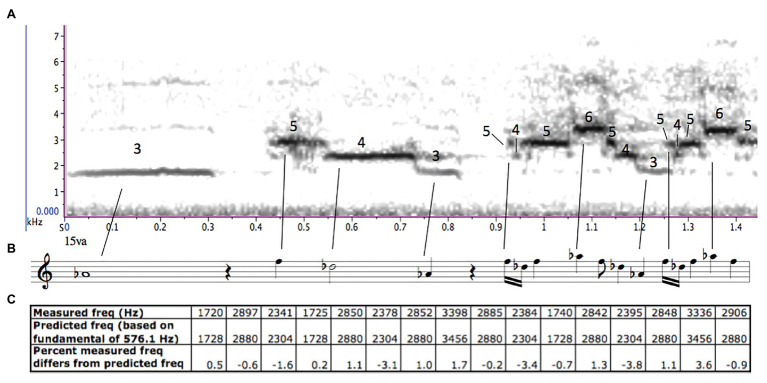
**(A)** A spectrogram of a hermit thrush song (Borror Laboratory of Ornithology), created on RavenPro 1.5 (Center for Conservation Bioacoustics, 2014), with the harmonics labelled above the sustained pitches. **(B)** An approximate musical transcription of the song. **(C)** The measured frequencies above the predicted unsung fundamental, compared with what the frequencies would be if the hermit thrush were singing perfectly in tune above that fundamental.

We began our research by listening to hermit thrush songs as we would listen to music, searching for any patterns or processes that might give insight into the structure of the songs. After first listening to hermit thrush recordings at full speed, we slowed them by a factor of four (using the software Raven Pro) to bring them into a more comfortable frequency range and tempo for human auditory processing. We heard immediately that most of the song-types seemed to follow the overtone (or harmonic) series (the series of frequencies in which each frequency is an integer multiple of an underlying fundamental). The birds never sang the fundamental of the series (which would be too low for a bird of that size to produce), but for each overtone-based song-type, all the pitches in the song (except the introductory whistle) were drawn from harmonics three through 12 above an unvoiced fundamental (**c, d,** and **e;** Doolittle et al., 2014). Our finding is similar to Cheney’s perception that the hermit thrush uses “thirds, fourths, fifths, and sometimes a whole octave” in its song, as all of these intervals can be found within the overtone series (as the ratios 6/5 and 5/4, 4/3, 3/2, and 2/1) – though ours goes further, in describing not only the intervals used, but also the relationship they have with each other. The unvoiced fundamental was different for each song-type, which likely accounts for the earlier listeners’ perception that the songs were following “harmonic progressions” or were in different “keys” (d; Smith, 1903; Mathews, 1904; Oldys, 1913; Figure 5).

We were not the first to hear that many hermit thrush song-types are constructed from the overtone series over different fundamentals – naturalist Ingraham (1938, p. 618), too, had mentioned in passing that hermit thrush song consists of a “piling up of overtones on shifting fundamentals,” without any further evidence or elaboration. But until now his observation had gone unnoticed beside the more colourful claims about hermit thrush song as an exemplar of either Western classical or “primitive” music.

As a musician, I was used to relying on my ear, and trusting my intuition when it came to determining which patterns were present and structurally significant in a piece of music. I was also used to reading papers, or indeed entire books, analysing a single piece of music. I was ready to publish immediately! But my scientific collaborators held me back. We “knew” that we had heard the harmonic series in hermit thrush song, but similarly, Oldys had “known” that it was based on the principles of Western classical music and Wing had “known” that it was based on the pentatonic scale. We were confident in our musical insight, but we needed to do more to make sure we were not just projecting patterns we were familiar with onto hermit thrush song, as some of the earlier zoomuicologists had done. In order to see whether our intuition would be supported by more rigorous analysis of data, we gathered recordings of 114 different hermit thrush song-types, sung by 14 hermit thrushes (Borror Laboratory of Bioacoustics, 2014; Colver, 2014; Krause, 2014). From these, we selected the 71 song-types that had 10 or more steady-pitch notes, and used the sound analysis software Praat (Boersma and Weenink, 1992-2010) to measure the frequencies of all the steady pitches. We then used both a Bayesian generative model (created by Endres) and a linear regression model (created by Gingras) to analyse the measured frequencies in each song. Both models independently confirmed our aural impression, that about 70% of the song-types follow the overtone series (**c, d;** Doolittle et al., 2014). Of the song-types that did not follow the overtone series, many used large numbers of noises and/or sliding pitches, suggesting that of the song-types consisting exclusively of steady-pitch sounds, substantially more than 70% were based on the overtone series. By contrast, in our control group of 1,000 “songs” generated from random pitches following a similar distribution to the hermit thrush songs, fewer than 5% followed the overtone series (Doolittle et al., 2014).

The overtone theory fits the data better than the pentatonic theory. The hermit thrushes showed no preference for a using five-note collection of pitches (over three, four, or six-note collections), and the pitches used follow a harmonic rather than scalar distribution – that is, the intervals sound closer together as they get higher, rather than being fairly equally distributed across an octave and repeated at each octave (**c**). And just as importantly, the overtone theory is plausible. While many cultures have some music which uses only five pitches, there is no culturally independent “pentatonic scale”: each culture that uses five note scales constructs, conceptualises, and/or uses them very differently. In other words, while there may be multiple kinds of pentatonic scale, there is no such thing as “the pentatonic scale.” The overtone series, on the other hand, is a physical phenomenon, present in many kinds of pitched sound, both natural and human-made. Many human musical cultures derive scales, melodies, or harmonies from the overtone series (Trehub, 2000), but there is nothing inherently human, or culturally specific, about the presence of the overtone series itself. Because many kinds of human scales – including some formations of the pentatonic, major, and minor scales – can be constructed using intervals from the overtone series, it is easy to understand why earlier listeners, without the benefit of recording equipment or analysis software, mapped what they heard onto what they were already familiar with. It is harder to understand why the myth of hermit thrush pentatonic scales has persisted for so long when there has never been any evidence that it is true!

Our finding is significant from both a musical and a biological point of view. The use of the overtone series in itself is neither a necessary nor a sufficient condition for something to be considered music: plenty of musical cultures give no special attention to the overtone series, and the overtone series can be heard in many pitched sounds, whether or not they are used in music. But as humans coming from a culture in which the overtone series does play an important organisational role, we were fascinated to hear how another species, too, structures its songs using some of the same sonic building material as we do. From a socio-musicological point of view, it was also interesting to discover the underlying pitch structure that had suggested so many different interpretations to people listening to it with such different sets of expectations. From a biological perspective, the presence of the overtone series opens up tantalising questions about commonalities of song construction and auditory processing between two such distantly related species. Does the hermit thrush use the overtone series because it is easier or more pleasurable to produce the pitches, because it makes the song easier to remember, or because it makes it easier to judge the quality of the song? Do “in-tune” hermit thrush songs correspond with the health and/or social status of the singer, as they do in the great tit (*Parus major*; Richner, 2016)? Are there any homologous auditory processing structures that are at the heart of humans’ and hermit thrushes’ shared interest in the overtone series? Did our two species converge on our use of the overtone series as a material for song construction for adaptive reasons? Or is just a coincidence that both humans and hermit thrushes are attracted to the overtone series? It is important to note, too, the limitations of our findings. Just because hermit thrushes base their songs on the overtone series does not mean other species do, and just because we have found the overtone series in hermit thrush song says nothing about whether or not the song is or is not music. Most importantly, we must disavow any kind of theory, which suggests music (or birdsong) that is based on the overtone series is somehow more “right” than any other kind of music (or birdsong), as this kind of thinking has in the past been used to support a Western classical music-centric devaluation of music’s that did not privilege overtone series-derived harmonic systems.

Finding the overtone series in hermit thrush song is of course only a first step towards understanding it. We heard other structural patterns too – song-types, which were used interchangeably (noticed also by Kroodsma, 2005, p. 256–267 and Nesbitt, 2020), and song-types which are transposed (frequency-shifted) versions of each other but are not used interchangeably, but as these were rare occurrences, we would need considerably more data to write about these observations in a scientific context. Further musical analysis may help us to understand the rhythmic structures, timbres, and non-pitched sounds that are also part of the song. Further biological observation may help us understand how hermit thrushes learn their songs and how they use them. My listing of nine key areas of potential overlap between human music and non-human animal song is not intended to be exhaustive, but rather suggests a number of potential starting points from which zoomusicological analysis may proceed. And as we have seen, most of these points are indeed embodied in hermit thrush song: it is vocally learned (**a**) and regionally varied (**b**); it uses a limited selection of pitched and non-pitched elements to create individually distinctive songs (**c, e,** and **f**), which are presented in a species-characteristic, semi-unpredictable order (**d** and **g**), primarily during breeding season (**h**). We do not yet know if the hermit thrush enjoys its song (**i**), but research on other songbirds (Kubikova et al., 2010; Stevenson et al., 2020) suggests that this appealing possibility may yet prove to be true.

In advocating for interdisciplinary research into hermit thrush song – and into animal song more generally – I do not mean to suggest that we should abandon disciplines altogether. Indeed, it is extremely valuable to be able to look at an animal song through as many disciplinary lenses as possible. We need to be able to listen to non-human animal song and make free associations with other things we have heard, including human music, without worrying about plausibility. We need to be able to observe behaviour and try to fit our observations in with what we know about the behaviour of other closely and distantly related species. We need to be able to look at raw data and try to figure out what patterns are present without trying to fit our observations in with our culturally-influenced expectations of what we might find. And we need to be open to historically-informed criticism, which situates our work in its historical and cultural context, and shows where we may be projecting our own worldview onto the object at hand, even when we think we are not. I look forward to reading a paper 30 years from now that shows where my collaborators and I, too, were influenced by prevailing cultural thought patterns even though we were trying to avoid this! Having access to the different methodologies and different analytical tools used in different fields is important, but equally important is combining different ways of working, asking different kinds of questions, and valuing different kinds of knowledge. After we have looked at animal songs through our separate disciplinary lenses, let us always remember to reconvene to see how our different perspectives can interrogate, challenge, and ultimately strengthen each other.

## Data Availability Statement

Publicly available datasets were analysed in this study.

## Author’s Note

This paper is based in part on an earlier essay, entitled *Music Theory is For the Birds*, which appeared in the conference book *Sound in the Land – Music and the Environment*, edited by Carol Ann Weaver, Doreen Helen Klassen, and Judith Klassen, and published by *The Conrad Grebel Review* 33, no. 2 (Spring, 2015, 238–248). This expanded and updated version appears here with permission of the original publisher.

## Author Contributions

The author confirms being the sole contributor of this work and has approved it for publication.

### Conflict of Interest

The author declares that the research was conducted in the absence of any commercial or financial relationships that could be construed as a potential conflict of interest.

## Footnotes

^2^No systematic study of Canyon Wren pitch relationships has yet been done, so I analysed two Canyon Wren songs from www.xeno-canto.org. The notes slide extensively, so I measured the lowest frequency of each. Whereas a chromatic scale would consist of a series of pitches 100 cents apart, one of the songs I analysed consisted of 11 pitches unequally distributed over a range of 1,956 cents (which averages to 178 cents apart), and the other of 15 pitches unequally distributed 1,678 cents (which averages to 112 cents apart). Even if the notes had been steady, measurable pitches that were distributed equally, they would not have approximated a chromatic scale.

^3^Some of these points of potential overlap between human music and animal song were first presented in Doolittle (2007) and/or Doolittle (2015).

^4^Indigenous peoples of North America have of course been aware of and knowledgeable about hermit thrush song since long before then, but would historically have shared their knowledge orally rather than in written documents.

^5^For further reading on whether the pentatonic scale can be considered a musical “universal,” see Sachs (1962), Nettl (1977), Agawu (2016), and others.

^6^Thank you to reviewer 2 for suggesting this particularly apt quotation.
